# Crystal structure of UbiX, an aromatic acid decarboxylase from the psychrophilic bacterium *Colwellia psychrerythraea* that undergoes FMN-induced conformational changes

**DOI:** 10.1038/srep08196

**Published:** 2015-02-03

**Authors:** Hackwon Do, Soo Jin Kim, Chang Woo Lee, Han-Woo Kim, Hyun Ho Park, Ho Min Kim, Hyun Park, HaJeung Park, Jun Hyuck Lee

**Affiliations:** 1 Division of Polar Life Sciences, Korea Polar Research Institute, Incheon 406-840, Republic of Korea; 2 Department of Polar Sciences, Korea University of Science and Technology, Incheon 406-840, Republic of Korea; 3 Graduate School of Medical Science and Engineering, Korea Advanced Institute of Science and Technology (KAIST), Daejeon 305-701, Republic of Korea; 4 Department of Biochemistry, School of Biotechnology and Graduate School of Biochemistry, Yeungnam University, Gyeongsan, Republic of Korea; 5 X-Ray Core, Scripps Florida, Jupiter, FL 33458, USA

## Abstract

The *ubiX* gene of *Colwellia psychrerythraea* strain 34H encodes a 3-octaprenyl-4-hydroxybenzoate carboxylase (CpsUbiX, UniProtKB code: Q489U8) that is involved in the third step of the ubiquinone biosynthesis pathway and harbors a flavin mononucleotide (FMN) as a potential cofactor. Here, we report the crystal structures of two forms of CpsUbiX: an FMN-bound wild type form and an FMN-unbound V47S mutant form. CpsUbiX is a dodecameric enzyme, and each monomer possesses a typical Rossmann-fold structure. The FMN-binding domain of UbiX is composed of three neighboring subunits. The highly conserved Gly15, Ser41, Val47, and Tyr171 residues play important roles in FMN binding. Structural comparison of the FMN-bound wild type form with the FMN-free form reveals a significant conformational difference in the C-terminal loop region (comprising residues 170–176 and 195–206). Subsequent computational modeling and liposome binding assay both suggest that the conformational flexibility observed in the C-terminal loops plays an important role in substrate and lipid bindings. The crystal structures presented in this work provide structural framework and insights into the catalytic mechanism of CpsUbiX.

Ubiquinone is a universal membrane-associated redox mediator present in most cells. It is an important component of the electron transport chain and is involved in aerobic cellular respiration^1^. Furthermore, in its reduced form, ubiquinone functions as an antioxidant in mammalian cells^2^. In prokaryotic organisms, several *ubi* gene clusters encoding a set of enzymes (UbiA, UbiB, UbiC, UbiD, UbiE, UbiF, UbiG, UbiH, and UbiX) produce ubiquinone from its precursor, chorismate^1^,^3^. Among them, UbiX and UbiD are isofunctional aromatic decarboxylases (EC 4. 1. 1.) that catalyze the third step of ubiquinone biosynthesis, in which 3-polyprenyl-4-hydroxylbenzoate is converted into 2-polyprenylphenol following the prenylation of 4-hydrobenzoate^4^. Despite being isofunctional, UbiX and UbiD share no sequence or structural similarities. Previous studies have shown that the expression of these two proteins is regulated by growth conditions and that both enzymes are required for the normal production of ubiquinone in the log phase of *Escherichia coli* cells^4^. Despite their biologically important functions in the electron transport system, limited information is available regarding their substrate recognition, specificity, and reaction mechanisms. This is partly because the native substrates of these proteins, the membrane-associated hydrophobic polyprenyl *p*-hydroxybenzoate (PPPHB), are water-insoluble, and this impedes *in vitro* enzyme activity assays as well as determination of the crystal structures of the proteins in complex with the substrates.

In a previous study, we purified recombinant *Colwellia psychrerythraea* 34H UbiX protein (CpsUbiX) in a flavin mononucleotide (FMN)-bound form from an *E. coli* expression system and successfully crystallized the purified protein^5^. Moreover, a V47S mutation resulted in complete loss of FMN binding, allowing us to produce an FMN-free form of CpsUbiX without affecting overall structure of the protein. In this study, we determined the crystal structures of FMN-bound (wild type) and FMN-free (V47S mutant) UbiX, at 1.9 and 1.76 Å resolutions, respectively. Although crystal structures of FMN-bound UbiX from *Pseudomonas aeruginosa* (PDB code: 3ZQU) and UbiX-like decarboxylase (Pad1) from *E. coli* (PDB code: 1SBZ) have been reported^6^,^7^, no structural information on the FMN-free form of UbiX is available to date. Comparison of the two crystal structures (FMN-bound and FMN-free CpsUbiX) revealed key residues involved in FMN binding as well as in the conformational changes associated with it. Notably, the C-terminal region (residues 195–206) underwent a significant conformational change following FMN binding. In addition, we have modeled a substrate bound structure based upon the idea that the labile C-terminal residues may contribute proper substrate binding site (and active site formation) only in the presence of the substrate. Liposome binding studies suggest that CpsUbiX associates with membrane and that FMN binding enhances the association. Therefore, we propose that the C-terminal region plays the role of a gatekeeper for FMN and substrate binding.

## Results and Discussion

### Overall structure of FMN-bound CpsUbiX

**Figure 1:**
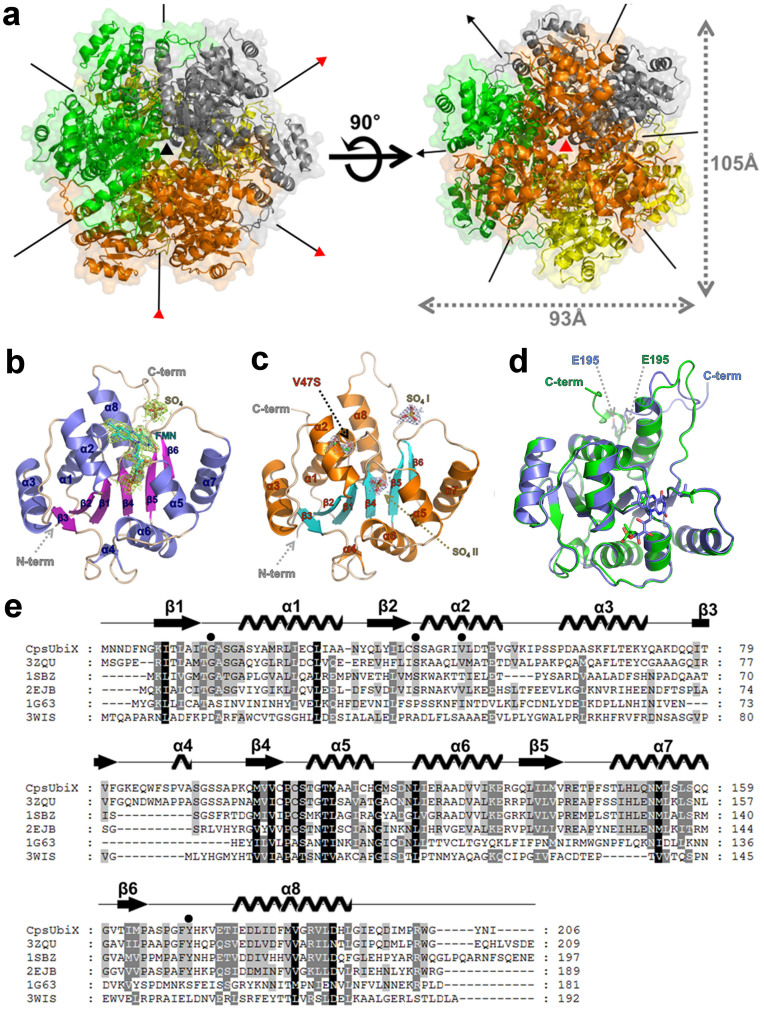
Crystal structure and sequential alignment of CpsUbiX. (a) Ribbon representation of the dodecameric crystal structure and two different three-fold axes of CpsUbiX. The dodecamer structure of CpsUbiX is composed of four trimers (colored in orange, green, yellow, and gray). Three-fold axis A is indicated with a red triangle while three-fold axis B is shown as a black triangle. (b) Ribbon diagram of the wild type (FMN-bound) CpsUbiX monomer. The bound FMN molecule and sulfate ion are shown as stick models. 2F_o_-F_c_ electron density maps (contoured at 1σ) are shown in yellow. The N- and C-termini are labeled. (c) Ribbon diagram of the V47S (FMN-free) mutant CpsUbiX monomer. The site of the mutation is labeled as V47S. The FMN-free structure contains two sulfate ions (SO_4_ I and SO_4_ II) (d) Structural superposition of wild type (FMN-bound form, slate blue) and V47S mutant (FMN-free form, green) of CpsUbiX. The C-terminal loop region (residue 195–205) shows a swing movement upon FMN binding. (e) Multiple sequence alignment of CpsUbiX (UniProtKB code: Q489U8) and other homologous proteins identified by a DALI search (PDB code: 3ZQU, UniProtKB code: Q9HX08; PDB code: 1SBZ, UniProtKB code: P69772; PDB code: 2EJB, UniProtKB code: O66811; PDB code: 1G63, UniProtKB code: P30197; and PDB code: 3WIS, UniProtKB code: Q13QT8)^6^,^7^,^25^,^26^,^27^. Secondary structural elements of CpsUbiX are indicated above the sequences. The residues (Gly15, Ser41, Val47, and Tyr171) that constitute the flavin mononucleotide (FMN)-binding site are indicated by filled circles. The multiple sequence alignment was performed using ClustalX and edited using GeneDoc^18^

Crystal structures of CpsUbiX, with or without the cofactor FMN, were determined to resolutions of 1.9 Å and 1.76 Å, respectively. FMN-bound CpsUbiX crystallized in the orthorhombic space group (*C*222_1_) with 6 monomers in the asymmetric unit, while FMN-free CpsUbiX crystallized in the cubic space group (*P*23) with one molecule in the asymmetric unit. The crystal structures of CpsUbiX are similar to those previously reported homologs of UbiX (PA4019 and Pad1) with regard to the overall fold and nature of intermolecular interactions between the subunits^6^,^7^. The monomer structure adopts a typical Rossmann-fold topology comprising eight α-helices (residues 19–32, 42–52, 60–71, 89–91, 107–115, 121–132, 147–159, and 177–192) and six β-strands (residues 8–13, 35–40, 78–81, 99–103, 135–140, and 162–165). The six parallel β-strands are ordered as β3-β2-β1-β4-β5-β6 and are surrounded by the amphipathic helices. Electron density for FMN could be seen clearly in the subunit interface region of the complex crystal structure (Fig. 1 and Supplemental Fig. S1).

**Figure 2:**
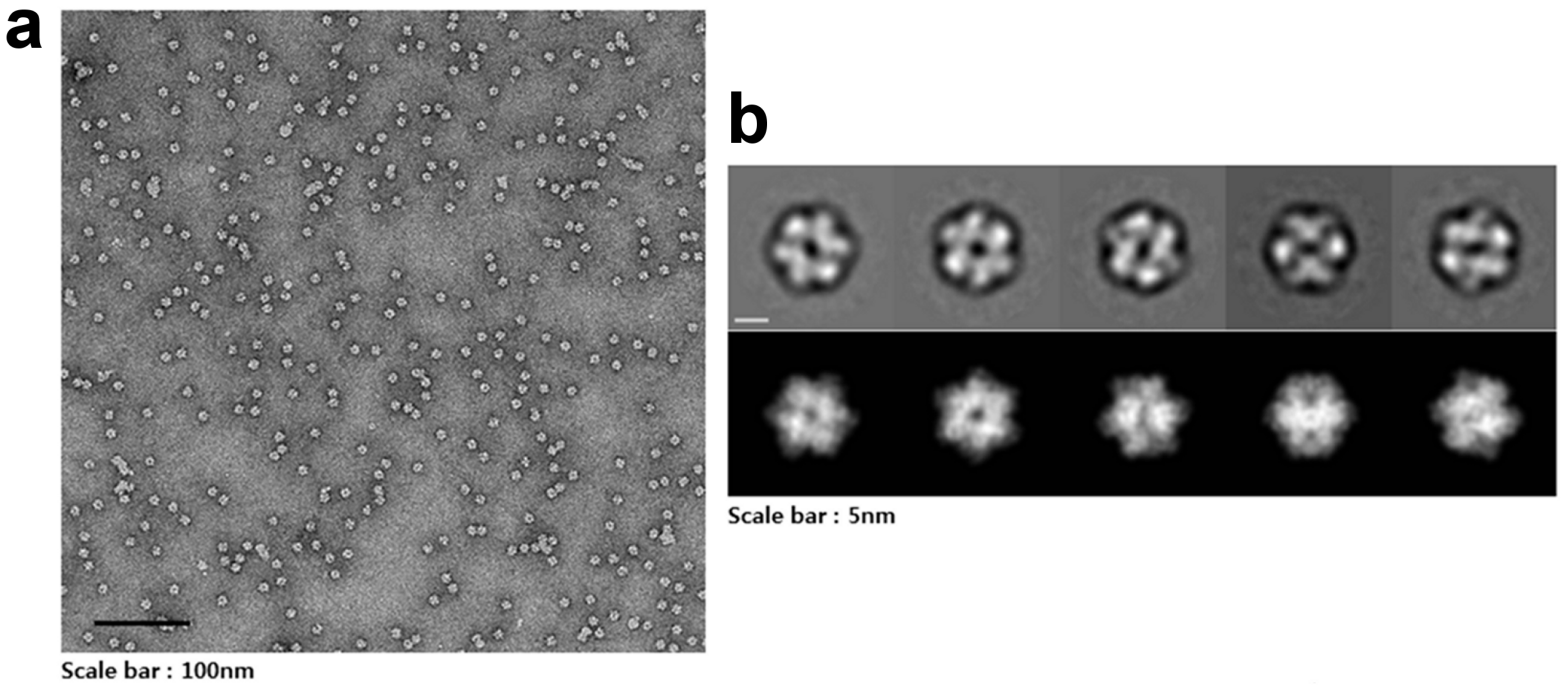
Dodecameric structure of CpsUbiX. (a) A typical micrograph depicting the CpsUbiX dodecamer. (b) Representative two-dimensional (2D) class averages of the CpsUbiX dodecamer and corresponding forward projection images. Negative-staining electron micrographs of the purified recombinant CpsUbiX showed ball-shaped structures approximately 10 nm in length. The scale bar is 5 nm.

The crystallographic symmetries of both crystal structures generate spherical dodecamers with dimensions of 105 Å × 93 Å × 95 Å (Fig. 1a). This is in good agreement with the results of analytical size-exclusion chromatography, where purified CpsUbiX showed an apparent molecular weight compatible with that of a dodecamer^5^. In an electron microscope (EM) study, negatively stained CpsUbiX specimens appeared as spherical particles with an average size of approximately 100 Å in diameter (Fig. 2). Therefore, the results of the EM study are in agreement with our dodecameric crystal structure of CpsUbiX.

The monomeric structure of CpsUbiX is stabilized by two different patches of inter-subunit contacts, namely patches 1 and 2, to generate a dodecamer. Patch 1 is formed by inter-helical hydrophobic interactions involving three hydrophobic residues, Leu150, Met153, and Leu154, at the α7-helix of one monomer and Pro144 and Phe145 located at the loop region between the β5-strand and α7-helix of another monomer. In addition, a hydrogen-bond network is involved in stabilizing the subunit interface around patch 1 (OG of Ser17 to N of Phe170, OG1 of Thr50 to O of Asn205, NH2 of Arg141 to O of Ser167, OG1 of Thr143 to O of Pro165, N of Phe145 to O of Ile163, and OG1 of Thr147 to OG of Ser157). The two interacting monomers are related to each other by a 2-fold symmetry. Patch 2 is formed by nine hydrogen bonds (O of His115 to ND2 of Asn152, NH1 of Arg124 to OE1 of Glu142, OD2 of Asp127 to NE2 of His149, NZ of Lys131 to OE2 of Glu142, NZ of Lys131 to O of Thr143, OE1 of Glu132 to NE2 of His191, OE2 of Glu132 to NH1 of Arg187, NH1 of Arg133 to O of Gly160, and NE2 of Gln159 to OE1 of Gln151) and salt bridges between neighboring subunits. The buried surface areas generated by patches 1 and 2 are 1650 Å^2^ and 1110 Å^2^, respectively (Supplemental Fig. S2 and Table S1)^8^,^9^.

**Figure 3:**
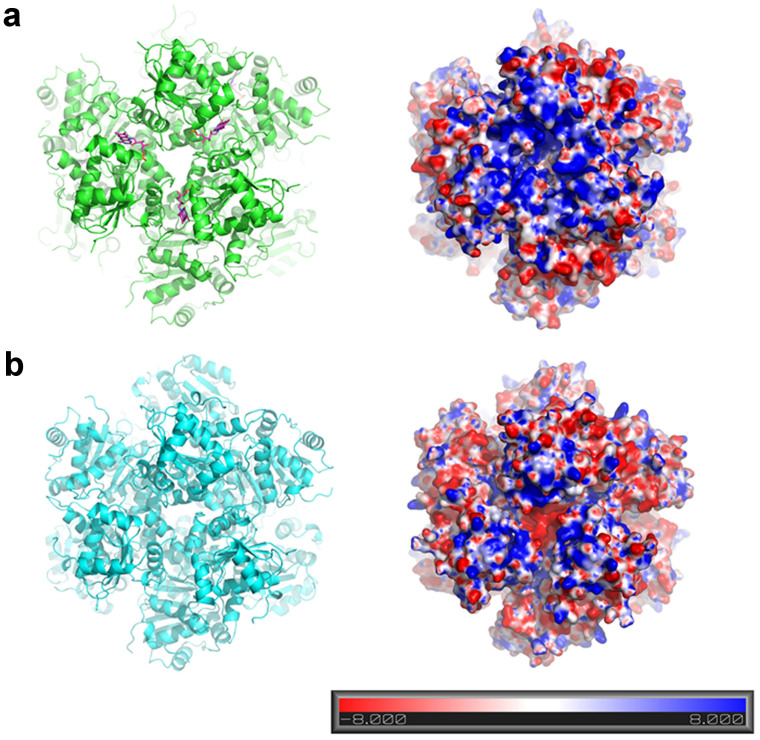
Electrostatic surface potential representations of CpsUbiX. (a) Cartoon view of dodecameric CpsUbiX (green) with bound FMN (purple sticks). Molecular surface representation of CpsUbiX shows a greater accumulation of positively charged residues (Lys45, Lys54, Pro60, Lys65, and Lys83) on the surface at the predicted membrane binding site. (b) Cartoon view of the dodecameric V47S mutant CpsUbiX (cyan) structure without FMN. Molecular surface representation of the V47S mutant CpsUbiX shows markedly reduced net positive surface charge. The molecular surfaces are colored according to electric potential. Blue and red represent positive and negative potentials (units of kT/e from −8 to +8), respectively. The orientation of the surface representation figure is identical to that illustrated in the left panel.

The dodecamer of CpsUbiX comprises two three-fold rotational axes: The three-fold axis A is located among the Asp119 residues from three different subunits, while the three-fold axis B is located at the tip of helix 7 (Fig. 1a). A vertical view of three-fold axis A revealed an entrance to the FMN binding sites. Electropotential surface analysis revealed that the dodecameric CpsUbiX contains a large positively charged patch around three-fold axis A, reflecting the presence of positively charged surface residues (Lys45, Lys54, Lys65, and Lys83; Fig. 3). The charged surface is more prominent in FMN-bound CpsUbiX. The patches are relatively flat and are located close to the FMN binding sites. Due to membrane embedded substrate, CpsUbiX may associate with membrane directly or indirectly though UbiA^10^. A homology model of *Colwellia psychrerythraea* 34H UbiA shows the cytosolic part of the protein near substrate binding site is negatively charged and the residues constructing the negative charged patch are highly conserved among orthologs of bacterial UbiAs. The positive patch described here could be an important feature for the membrane association.

### FMN-binding site

**Figure 4:**
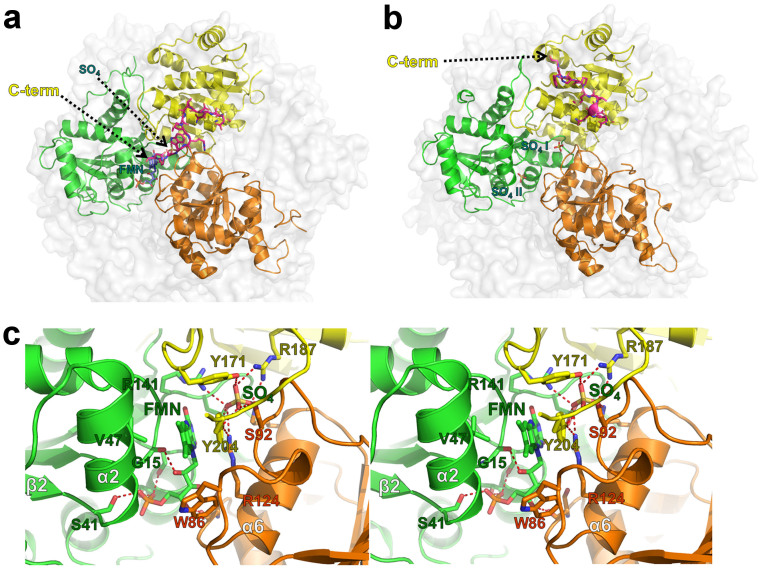
Structural comparison of the FMN-bound and FMN-free CpsUbiX structures. (a) The wild type CpsUbiX structure with a bound FMN molecule and one sulfate ion in the groove formed by three neighboring subunits. The C-terminal region residues (resi 195–205) of yellow colored subunit are shown in magenta for clarity. (b) The V47S mutant CpsUbiX structure with two sulfate ions (SO_4_ I and SO_4_ II) in the groove formed by three neighboring subunits. (c) Stereo view of the FMN-binding site. The FMN and interacting residues are shown as stick models. Notably, the C-terminal region residues (Tyr171 and Tyr204) are involved in FMN binding, but the region has a different conformation in the V47S mutant (FMN-free) structure.

FMN is bound to a positively charged groove in the subunit interface formed by three neighboring protomers. This suggests that the dodecameric organization of UbiX is necessary if FMN is directly involved in decarboxylation reaction. The FMN-binding site is surrounded by the α2 and α6 helices and the C-terminal loop region from another subunits (Fig. 4). The phosphoribityl group of FMN is buried deeply in the groove, whereas the isoalloxazine ring remains partially accessible to the solvent. In addition, a sulfate ion (SO_4_, Fig. 1b) is bound near the FMN isoalloxazine ring, which acts to stabilize the interactions between the subunits. The C-terminal residue, Tyr204, forms a stacking interaction with FMN. In addition, the hydrophobic residues, Val47, Ile46, Trp86, and Tyr171 delimit the hydrophobic part of the isoalloxazine moiety of FMN. The phosphate group of FMN forms three hydrogen bonds with Ser41, Ser106, and Thr109 (Fig. 4c). The phosphate-binding site in FMN-free UbiX is occupied by a sulfate ion (SO_4_ II, Fig. 1c).

A sulfate-binding site in crystal structures is often indicative of a phosphate-binding site in the physiological state of the protein. Because the substrate of CpsUbiX, PPPHB, is membrane-embedded, the decarboxylation reaction is expected to occur at the membrane surface. Therefore, in addition to the charge-dependent interactions described above, CpsUbiX might anchor on the membrane through a phospholipid with a phospho-head group such as phosphatidic acid and phosphatidylglycerophosphate using the sulfate-binding site near the FMN isoalloxazine ring (Fig. 1b). Alternatively, the sulfate-binding site could serve as a binding site for negatively charged carboxylate group of *p*-hydroxybenzoate (PHB) moiety. However, rough estimate shows such binding would expose large portion of polyprenyl to the solvent and hydrophilic side chains as the sulfate-binding site is located deep in the protein.

### Structural comparison between FMN-bound (wild type) and FMN-free (V47S mutant) CpsUbiX

Our previous spectroscopic analysis indicated that the V47S single mutation destabilized FMN binding and produced a FMN-free form of CpsUbiX^5^. Here, we confirmed that electron density corresponding to FMN was absent in the crystal structure of the V47S mutant (Fig. 1c). Val47 is located on the α2 helix, which extends from the β2 strand of the central β-sheet and makes van der Waals interactions with the dimethyl phenyl moiety of isoalloxazine. Therefore, mutating the Val47 to Ser significantly affected this hydrophobic interaction, resulting in the mutant protein having a low affinity toward FMN. Structural comparison of the FMN-bound and FMN-free forms revealed no significant change in the overall structure (root mean square deviation of 0.48 Å over 189 C_α_ atoms), with the exception of residues ranging from Phe170 to Thr176 and the C-terminal loop (residues 195–206). In contrast to FMN-free CpsUbiX, the FMN-bound CpsUbiX possesses a sharp turn at Ile198, causing the C-terminal end to point toward the interface between neighboring monomers, where FMN is bound (Fig. 4a and 4b). The C-terminal structure is also stabilized by Tyr204 through stacking interactions with FMN. On the other hand, the C-terminus is parallel to α7 in FMN-free CpsUbiX and is stabilized by Met199 and Trp202 through hydrophobic and hydrogen bond interactions. Tyr204 is exposed to the bulk solvent in the FMN-free CpsUbiX.

In contrast to the FMN-bound CpsUbiX, the structure of the C-terminal region of the FMN-free CpsUbiX extends into a β-sheet region of the core domain with the conformational difference starting at the Glu195 residue (Fig. 1d). The Gly203 residue shows a large swing movement of 26 Å at its C_α_ atom. As a result, the C-terminal region makes intramolecular interactions with the α2 and α8 helices. In addition to the swing movement of the C-terminal loop region, a conformational drift occurs in the loop region comprising residues 170–176. In FMN-bound CpsUbiX, Tyr171 makes edge-to-face hydrophobic interactions with Tyr204, which is stacked against FMN. Loss of this interaction in FMN-free CpsUbiX causes the residues His172 to Val174 to be relaxed and the Phe170–Thr176 loop region to drift towards the neighboring molecule (Supplemental Fig. S3a). This in turn leads to the formation of a large solvent channel connected to the putative active site in the FMN-free form. Until now, no FMN-free structures of UbiX have been described, and hence, the dynamics of the C-terminal and 170–176 loop have remained largely unknown for these UbiX homologues.

### Substrate binding model

AtHAL3, a 4-phosphopanthotenoyl cysteine (PPC) decarboxylase of *Arabidopsis thaliana,* is a rare example of an FMN-dependent decarboxylase for which the reaction intermediate-bound structure has been disclosed (PMID: 12614618). The ene-thiol reaction intermediate (PCO) is bound in a shallow surface pocket formed by a folded loop (residues Lys171–Tyr178) near the FMN-binding site (Supplemental Fig. S3). Comparison of this structure with that of FMN-bound CpsUbiX revealed highly conserved structural features, including the overall monomeric structure, architecture of the FMN-binding site, and oligomerization interfaces. There are also notable differences. First, there is no structural conservation between CpsUbiX and AtHAL3 at the substrate-binding site, clearly because these two proteins bind different substrates. Furthermore, there is a difference in the overall structures of CpsUbiX and AtHAL3 at the loop between β6 and α8. In CpsUbiX, the loop is a part of patch 1, which interacts with a neighboring residue (residues Ser17, Gly18, Glu51, Arg141, Thr143, Ala166, Ser167, and Ile181). Superposition of AtHAL3 (PDB ID: 1MVN) with CpsUbiX revealed that the same loop is twisted towards its own FMN, concurrently shaping a binding site for the substrate (Supplemental Fig. S3)^11^.

**Figure 5:**
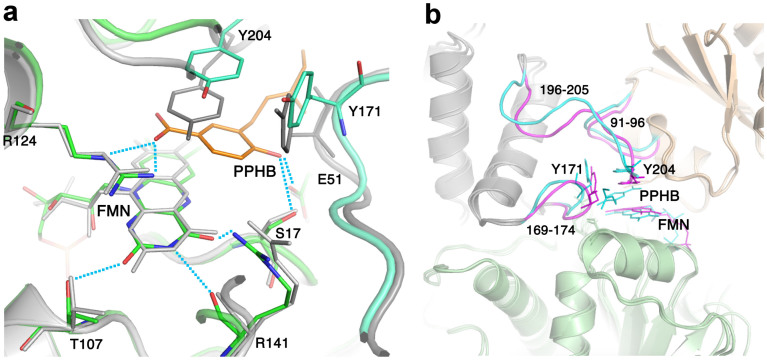
Overlay view of the CpsUbiX model bound to 3-prenyl-*p*-hydroxybenzoate (PPHB) and FMN and the crystal structure of CpsUibX-FMN. (a) PPHB is intercalated between the isoalloxazine ring of FMN and Tyr204. Key residues that make hydrogen bond (H-bond) interactions with FMN are shown as sticks. H-bonds are represented as dashed lines. The crystal structure and the model are colored grey and green, respectively. PPHB is represented as a orange stick. (b) Comparison of the loop regions that went through reorganization after the substrate binding. The reorganized loops of PPHB-bound modeled and the equivalent loops of the crystal structure are colored cyan and magenta, respectively, and the residue ranges are written.

Careful inspection of the CpsUbiX structure did not reveal a distinct pocket near the FMN for the substrate to bind. This indicates that a substrate-induced conformational change is necessary for the protein to generate a binding pocket for PHB. Moreover, such a pocket is expected to be a shallow or folded loop structure, similar to that seen in AtHAL3, to minimize entropic penalty. Using a steered molecular dynamics (SMD) approach, we investigated substrate entry and binding in FMN-bound CpsUbiX. Large motions observed in the loop residues ranging from Phe170 to Thr176, and in residues ranging from Glu195 to the C-terminus could be utilized for substrate binding. Therefore, 3-prenyl *p*-hydroxybenzoate (PPHB) was pulled toward the loop residues from the bulk solvent region to scan ligand-entry site. The result showed the steered ligand entered through the gap between residues Tyr171 and Ans205. The substrate-bound CpsUbiX model did not show any significant structural change in either the bound FMN or the protein except the regions that accommodate substrate entry (residue ranges Gly169–Val174 and Gln196–Asn205 of a monomer and Ala91–Ala96 of another monomer) (Fig. 5a). The hydrogen bonds between the FMN and protein residues were also preserved mostly. The modeled PPHB displaced the side chain of Tyr204 and stacked between that and isoalloxazine ring of FMN. The distance between C_5_ of FMN to C_1_ of PPHB was 3.5 Å. The displaced Tyr204 still preserved the edge-face interaction with Tyr171. Carboxylate of PPHB made charge interaction with the side chain of Arg124 while the hydroxyl group made hydrogen bond to the side chains of Ser17 and Glu51.(Fig. 5b).

No information regarding the enzyme activity of CpsUbiX or its orthologs is available. Therefore, interpreting the current simulation result would be impractical. However, our model shows the substrate can stack close proximity to FMN suggesting possible direct involvement of the cofactor in decarboxylation reaction. Once prenylated, only the PHB moiety of PPPHB is exposed to the membrane surface, while the hydrophobic polyprenyl group is embedded in the membrane. Therefore, the substrate-binding site of CpsUbiX would ideally face the membrane in order to minimize exposure of the polyprenyl group and PHB moiety. The proposed substrate-entry site is parallel to the three-fold axis A, which has a prominent positively charged patch on the enzyme. It also reduces the distance between the membrane surface and the FMN site of the enzyme.

### CpsUbiX membrane interaction

**Figure 6:**
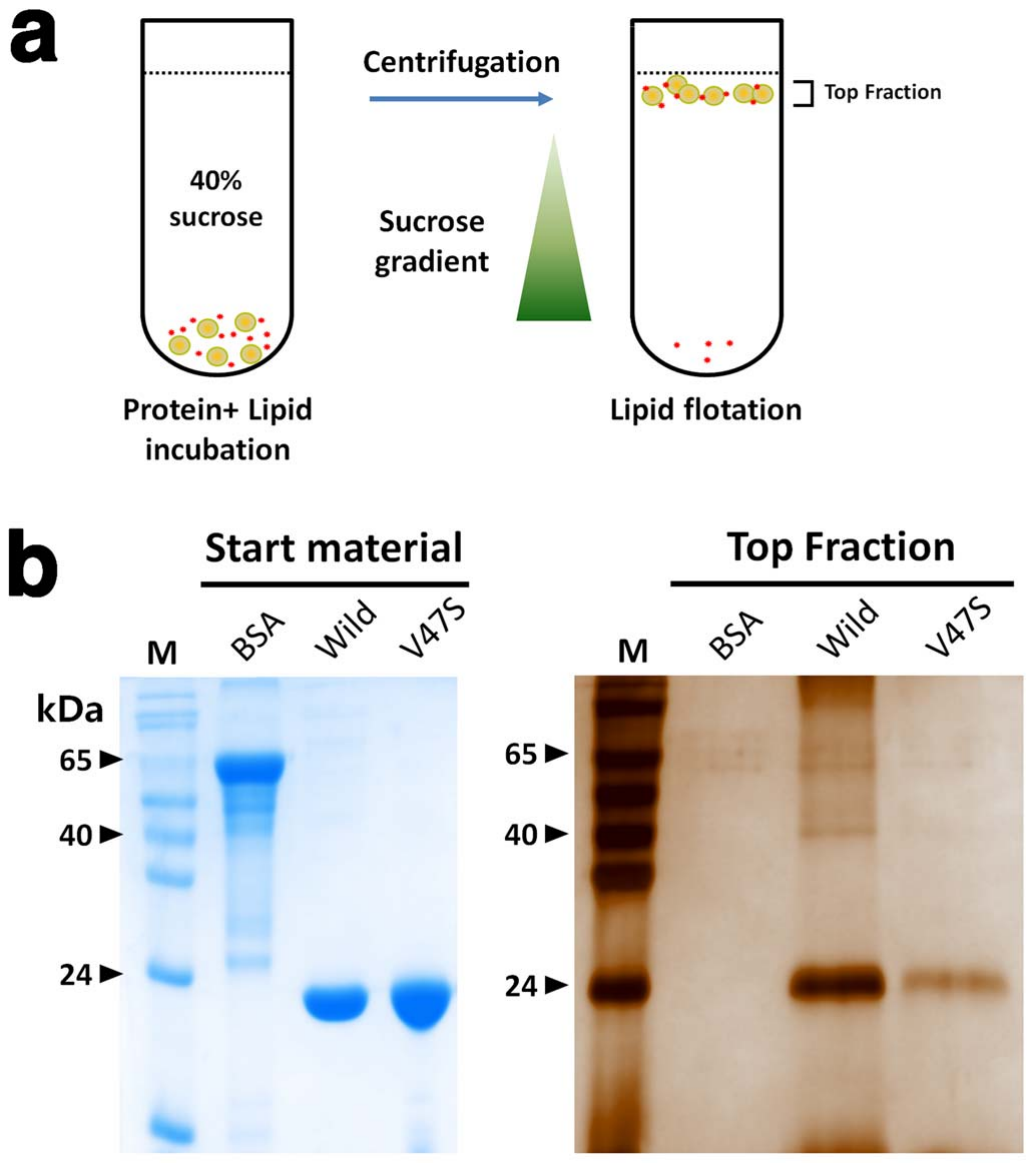
Membrane interaction assay. (a) Schematic diagram outlining the steps involved in the lipid flotation assay of CpsUbiX. A mixture containing liposomes (phosphatidylcholine [PC]:phosphatidylglycerol [PG], 2:1) and protein (wild type, V47S mutant, or BSA [negative control]) was incubated for 20 min and floated through a sucrose gradient by centrifugation. (b) The floated fraction was analyzed by sodium dodecyl sulfate-polyacrylamide gel electrophoresis (SDS-PAGE; stained by silver-staining). Bovine serum albumin (BSA) was used as a negative control. The results of the experiment revealed that wild type CpsUbiX strongly associated with the prepared liposomes, whereas the V47S mutant (FMN-free) bound to liposomes with weak affinity compared to the wild type.

The substrate of CpsUbiX is present at the inner leaflet of the bacterial inner membrane^12^. One possibility for optimal substrate binding is that CpsUbiX recognizes the membrane-embedded substrate without directly interacting membrane. However, the enzymatic reaction would be more efficient if CpsUbiX were concentrated on the surface of the membrane, close to where the substrate is located. Surface analyses of CpsUbiXs revealed that the protein might interact with the cytosolic membrane via its positively charged surface in a charge-dependent manner. We tested whether CpsUbiX directly interacts with membranes using liposome-binding assays. To this end, we performed a lipid flotation assay using liposomes that were prepared to mimic the inner leaflet of the bacterial cytosolic membrane (Fig. 6a)^13^. The experiments revealed that the wild type CpsUbiX strongly associated with the prepared liposomes. In contrast, the V47S mutant (FMN-free) bound to the liposomes weakly, indicating that an FMN-dependent structural change in CpsUbiX is required for efficient liposome binding (Fig. 6b). Electropotential surface analysis showed that FMN-free CpsUbiX possesses a less basic surface charge distribution. These results suggest that CpsUbiX directly associates with the membrane, and the structural change associated with FMN binding is important for its membrane targeting. Furthermore, our PPHB-bound model structure of CpsUbiX revealed that the binding pocket for the substrate is shallow, suggesting low binding affinity for the substrate. Therefore, membrane association of CpsUbiX might compensate for the low substrate binding affinity by accumulating the enzyme near the substrate. It is also intriguing to note that no enzyme activity has been demonstrated for UbiX in biochemical assays, and this may be because of the lack of membrane-embedded substrates in the design of the assay. Further studies are needed to determine the activity and kinetic parameters of UbiX toward hydrophobic substrates.

## Conclusion

This study provides two new crystal structures of CpsUbiX in a FMN-bound and FMN-free state. In addition, we modeled a substrate-bound structure based on the two crystal structures. Large structural changes were seen in C-terminal region upon FMN-binding; the co-factor binding led to a reorganization of the loop residues (residues 170–176) and the C-terminus (residues 195–206). FMN binding induces a swinging of the C-terminal residues. Our modeling of the substrate-bound state was based on the idea that the labile C-terminal residues may contribute to the formation of the substrate-binding site (and active site) before the enzymatic reaction occurs. The result shows that the substrate is stacked against isoalloxazine ring of FMN suggesting FMN is involved in decarboxylation reaction. Further, we demonstrated that CpsUbiX is associated with the membrane and that the reorganization of the C-terminal residues upon FMN binding appears to be important for its association with the membrane. To date, no enzyme activity of UbiX has been detected in biochemical assays. This might be because of the lack of a suitable membrane-associated substrate in these assays. Our crystal structures and model might aid future studies on the reaction and substrate-binding mechanisms of CpsUbiX.

## Methods

### Cloning, expression, and purification of CpsUbiX

The methods for expressing and purifying recombinant CpsUbiX (UniProt accession no.: Q489U8) have been described previously^5^. Briefly, the full-length *CpsUbiX* gene (residues 1–206) was obtained from genomic DNA isolated from *C. psychrerythraea* 34H (ATCC BAA-681) by polymerase chain reaction (PCR) and cloned between the *Nde*I and *Xho*I sites of the pET-28a(+) vector. The resulting plasmid, confirmed by DNA sequencing (Macrogen, Korea), was used to transform competent *E. coli* BL21 (DE3) cells. A single transformed colony was selected and cultured overnight at 310 K. Forty milliliters of this culture was inoculated into 4 L of Luria-Bertani medium containing kanamycin (100 mg/mL), and the cells were grown at 310 K. Expression of CpsUbiX was induced by the addition of isopropyl β-d-1-thiogalactopyranoside (IPTG) to a concentration of 0.1 mM when the optical density at 600 nm (OD_600_) reached 0.6. At this point, the temperature was decreased to 298 K and the culture was placed in a shaking incubator for 18 h. The cells were harvested by centrifugation and resuspended in a buffer (50 mM sodium phosphate buffer, pH 8.0, 300 mM NaCl, and 5 mM imidazole) supplemented with 0.2 mg/mL lysozyme and 0.5 mM phenylmethylsulfonyl fluoride (PMSF). The cells were lysed by sonication and the cellular debris was separated by centrifugation at 16,000 × *g* for 1 h at 277 K. The supernatant was poured into a gravity-flow column packed with 10 mL Ni-NTA agarose resin (Thermo Scientific, Rockford, USA) and subjected to bead binding. The column was washed with 200 mL of a washing solution containing 50 mM sodium phosphate buffer, pH 8.0, 300 mM NaCl, and 20 mM imidazole. The protein was then eluted in 30 mL of washing solution containing 300 mM imidazole. The His-tag was cleaved by treatment with thrombin overnight, and the mixture was loaded on a Superdex-200 HiLoad 16/60 size-exclusion column (GE Healthcare) in buffer A containing 20 mM Tris-HCl, pH 8.0, 150 mM NaCl and 1 mM dithiothreitol (DTT). Finally, the purified protein was concentrated to ~21 mg·mL^−1^. The extinction coefficient of the protein at 280 nm was assumed to be 19,940 L·cm^−1^·mol^−1^. The V47S mutant CpsUbiX^5^ was expressed and purified using methods similar to those described above. All purification steps were performed at 277 K and the final protein obtained was stored at 193 K.

### Crystallization and data collection

Wild type CpsUbiX crystals grew in hanging drops containing reservoir buffer (200 mM sodium chloride, 100 mM potassium phosphate monobasic/sodium phosphate dibasic, pH 5.8, and 11% [w/v] polyethylene glycol [PEG]-8000) and protein solution (21 mg/mL in buffer A) mixed in a 1:1 volume ratio. The wild type dataset at 1.9 Å resolution was collected at 100 K at beamline BL-7A, Pohang, Korea. To obtain crystals of the V47S mutant form, 1 μL of the reservoir solution (0.1 M tri-sodium citrate, pH 5.4, 0.5 M ammonium sulfate, and 1.2 M lithium sulfate) was mixed with 1 μL of concentrated (25 mg/mL) protein sample and allowed to equilibrate with 0.5 mL of the well solution at 293 K. A native data set from the V47S crystals was collected at beam line BL-5C, Pohang, Korea. The high resolution limit of diffraction data was determined by considering the balance of I/σ(I), completeness, R_merge_ and CC1/2^14^ values in the highest resolution shell.

### Structure determination and refinement

In preliminary X-ray crystallographic studies, we determined the structure of wild type CpsUbiX to a resolution of 1.9 Å by molecular replacement using the structure of FMN-bound UbiX from *P. aeruginosa* (PDB entry: 3ZQU; sequence identity, 65%) as the model^6^. The crystals belonged to the space group *C*222_1_ with unit cell dimensions of *a* = 107.2 Å, *b* = 141.9 Å, and *c* = 170 Å. The structure of V47S CpsUbiX was solved by molecular replacement using MOLREP in the space group *P*23^15^. Refinement of the wild type and V47S mutant forms of CpsUbiX were performed using Refmac5, interspersed with manual model building using COOT^16^. The final model was checked using MolProbity (http://molprobity.biochem.duke.edu) and the sfcheck program of the CCP4 suite^9^,^17^. The refinement statistics and final model quality are listed in Table 1. Sequence alignments were performed using ClustalX2 and graphed using GeneDoc^18^. Figures were prepared using Pymol (www.pymol.org). The crystallographic data for FMN-bound (wild type) and FMN-free (V47S mutant) CpsUbiXs were deposited in the Protein Data Bank with accession codes 4RHE and 4RHF, respectively.

### Electron microscopy

Purified CpsUbiX (2 μL; concentration, 23 μg/mL) was applied to a glow-discharged copper grid covered with a continuous carbon film. The sample was negatively stained with 0.75% (w/v) uranyl formate and was examined under a Tecnai T120 microscope operated at 120 kV. Images were collected at a nominal magnification of 64,000× with defocuses ranging from −0.9 μm to −1.2 μm using an FEI Eagle 4 K × 4 K CCD camera (1.64 Å/pixel, FEI Eindhoven, The Netherlands).

### Image processing

Particles were semi-automatically selected using the EMAN2 boxer program, and bad particles were excluded manually^18^,^19^. Selected particles (16,184) were subjected to two-dimensional (2D) reference-free alignment, multivariate statistical analysis (MSA), and MSA classification. The 2D reference-free image analysis was performed using IMAGIC^19^. Five classes were compared to forward projection images of the dodecamer model.

### Preparation of liposomes

Lipid was purchased from Avanti Polar Lipids (http://avantilipids.com). Large unilamellar vesicles (liposomes) for the binding assay were prepared according to the manufacturer's protocol. Briefly, a mixture of phosphatidylcholine (PC) and phosphatidylglycerol (PG) at a ratio of 2:1 (w/w) was dried using nitrogen gas followed by a vacuum pump system in a siliconized round bottom tube. Liposomes were produced by adding phosphate-buffered saline (PBS) into the dried lipid layer, and the mixture was vortexed for at least 1 h. The vesicles were extruded 20 times through a 100-nm filter using an Avanti Extruder (Avanti Polar Lipids) and used immediately.

### Lipid floating assay

Protein binding to liposomes was investigated by floatation assays as described previously^13^. Thirty microliters of 0.15 mM (wild-type CpsUbiX) or 20 μL of 0.2 mM (V47S mutant CpsUbiX) of protein were incubated with 150 μL of liposomes at room temperature for 20 min. The protein-liposome mixture was mixed with sucrose (final concentration, 50%) and overlaid with 4 mL of a 40%-sucrose floating cushion in 14-mm × 95-mm Ultra-clear centrifuge tubes (Beckman coulter). The top surface was covered with PBS (200 μL). The centrifugation was performed at 30,000 rpm for 3.5 h at 298 K in a SW 40 Ti rotor using an Optima™ XE 90K Ultracentrifuge (Beckman Coulter). 500 μL from the top of the cushion was collected and precipitated by adding 20% trichloroacetic acid (TCA) to the mixture. The pellets were washed two times with cold acetone, resolubilized in 100 μL of 2 × SDS sample buffer and then the sample was analyzed by SDS-PAGE followed by silver staining to identify the amount of liposome-bound protein.

### Computational modeling

A trimeric CpsUbiX (around the FMN site) was modeled from the crystal structure of FMN-bound CpsUbiX for molecular dynamics simulation by deleting the sulfates, water molecules, uninvolved protein chains, and FMNs. Coordinates for PPHB were generated using Maestro (Schrodinger, LLC, New York) and introduced in the CpsUbiX structure near the FMN, approximately 8 Å away from the protein. Orientational and positional restraints were applied to the protein coordinates except the residue ranges from Phe87 to Pro97, Pro165 to Glu179, and Glu195 to Asn205 which are in close proximity to PPHB. The modeled structure was then solvated with TIP3 water and 150 mM NaCl using VMD^20^. Molecular dynamics simulation was performed using NAMD 2.9 with the CHARMM27 force field^21^,^22^. Prior to MD simulations, the system was pre-equilibrated by unrestrained minimization, followed by relaxation of the system by incremental heating and annealing, and finally equilibrated for 20 ps at a constant temperature (300 K) and pressure (1.01325 bar). The pull vector for substrate entry was calculated between the C_4_ of PPHB and Cα of Leu148 of the FMN-bound monomer, and the substrate was pulled with a spring constant of 17 kcal/mol/A^2^ and a pull velocity of 0.001 Å/timestep for 40 ps. A snapshot of the steered molecular dynamics run was extracted when the carboxyl moiety of PPHB was closest to the FMN, and the structure was minimized using NAMD and Prime (Schrodinger, LLC, New York) for analysis^21^.

### Electropotential surface analysis

To analyze the electric potential of the protein surface, the coordinates of the dodecameric structure of CpsUbiX were converted into the PQR format using the PDB2PQR server^23^. Electropotential surface analysis was performed using the APBS module in PyMOL^24^. The linearized Poisson-Boltzmann equation was used at a systemic temperature of 310 K for the analysis.

## Author Contributions

H.J.P. and J.H.L. designed and supervised the project. H.D. and C.W.L. performed cloning, expression, and protein purification. H.D. and J.H.L. crystallized, collected X-ray data and solved the protein structures. S.J.K. and H.M.K. performed the electron microscopy experiment. C.W.L., H.-W. K., H.P. and H.H.P. performed lipid floating assay. H.J.P. did the molecular dynamics simulation. H.D., H.J.P. and J.H.L. wrote the manuscript. All the authors discussed the results, commented on the manuscript, and approved the manuscript.

## Additional information

**Accession codes**: PDB i.d. numbers for the two structures are: 4RHE (FMN-bound wild-type CpsUbiX) and 4RHF (FMN-free V47S mutant CpsUbiX).

## Supplementary Material

Supplementary Information Supplementary data

## Figures and Tables

**Table 1:** Data collection and refinement statistics

Data set	Wild type (FMN-bound form)	V47S mutant (FMN-free form)
X-ray source	PAL 7A beam line	PAL 5C beam line
Space group	*C*222_1_	*P*23
Cell dimensions		
* a, b, c* (Å)	107.2, 141.9, 170.0	97.6, 97.6, 97.6
* α, β, γ* (°)	90.0, 90.0, 90.0	90.0, 90.0, 90.0
Wavelength (Å)	0.97934	0.97952
Resolution (Å)	50.0–1.90 (1.93–1.90)	48.81–1.76 (1.86–1.76)
Total reflections	698142	156791
Unique reflections	95103 (5078)	30485 (4430)
Average I/σ(I)	31.0 (5.7)	14.6 (7.0)
R_merge_^a^	0.116 (0.492)	0.081 (0.200)
CC1/2 (%)	98.6 (93.9)	99.7 (98.0)
Redundancy	7.3 (7.7)	5.1 (5.2)
Completeness (%)^b^	93.2 (100)	99.1 (99.6)

Refinement		
Resolution range (Å)	50.0–1.90 (1.95–1.90)	43.65–1.76 (1.81–1.76)
No. of reflections of working set	90030 (7054)	28946 (2130)
No. of reflections of test set	4813 (361)	1535 (96)
No. of amino acid residues	1212	200
No. of water molecules	263	50
R_cryst_ b	0.204 (0.230)	0.214 (0.229)
R_free_ c	0.260 (0.306)	0.243 (0.277)
R.m.s. bond length (Å)	0.019	0.019
R.m.s. bond angle (°)	1.987	1.973
Average B value (Å^2^) (protein)	30.53	25.386
Average B value (Å^2^) (solvent)	27.70	23.362
Average B-value (Å^2^) (FMNs)	30.69	
Ramachandran plot		
Most favored (%)	90.0	89.1
Additional allowed (%)	9.8	10.3
Generously allowed (%)	0.2	0.6
Disallowed (%)	0.0	0.0

aR_merge_ = | <I> − I | /<I>.

bR_cryst_ = | |Fo| − |Fc| | /|Fo|.

cR_free_ calculated with 5% of all reflections excluded from refinement stages using high-resolution data.

Values in parentheses refer to the highest resolution shells.
