# Supplementary material for: Crystal structure of UbiX, an aromatic acid decarboxylase from the psychrophilic bacterium Colwellia psychrerythraea that undergoes FMN-induced conformational changes
Source: Sci Rep. 2015 Feb 3;5:8196. doi: 10.1038/srep08196 (PMC4316190; doi:10.1038/srep08196)
Supplement: Supplementary Information — Supplementary data [file srep08196-s1.doc]

Supplemental Information for

**Crystal structure of UbiX, an aromatic acid decarboxylase from the psychrophilic bacterium *Colwellia psychrerythraea* that undergoes FMN-induced conformational changes**

Hackwon Do^1^, Soo Jin Kim^3^, Chang Woo Lee^1,2^, Han-Woo Kim^1^, Hyun Ho Park^4^, Ho Min Kim^3^, Hyun Park^1,2^, HaJeung Park^5*^ and Jun Hyuck Lee^1,2*^

^1^Division of Polar Life Sciences, Korea Polar Research Institute, Incheon 406-840, Republic of Korea

^2^Department of Polar Sciences, Korea University of Science and Technology, Incheon 406-840, Republic of Korea

^3^Graduate School of Medical Science and Engineering, Korea Advanced Institute of Science and Technology (KAIST), Daejeon 305-701, Republic of Korea

^4^Department of Biochemistry, School of Biotechnology and Graduate School of Biochemistry, Yeungnam University, Gyeongsan, Republic of Korea

^5^X-Ray Core, TRI, The Scripps Research Institute, Jupiter, FL 33458, USA.

Supplementary Figure S1


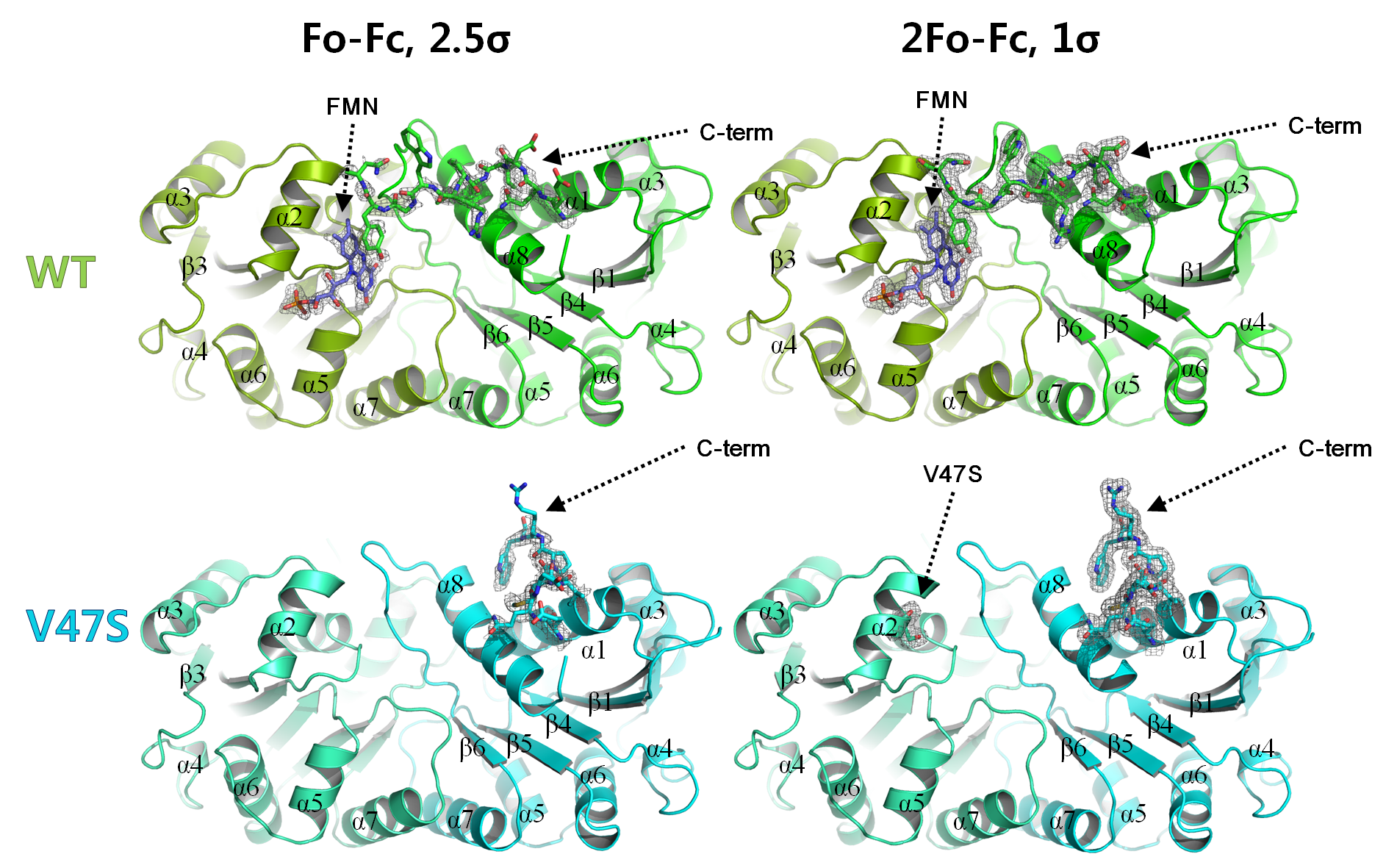


Figure S1. Conformational difference was observed in the C-terminal region of wild-type (upper panel) and V47S mutant (lower panel). The Fo-Fc omit map contoured at 2.5σ (in grey) was calculated excluding the residues 195-206, as shown in the left panel. In the right panel, the 2Fo-Fc map (contoured at 1σ) was shown after final modeling of the C-terminal loop and FMN. For clarity, only the dimers of wild-type and mutants were presented in different color codes. The V47S mutation results in the loss of FMN binding, and the mutant clearly shows significant conformational changes in the C-terminal region (lower panel).

Supplementary Figure S2


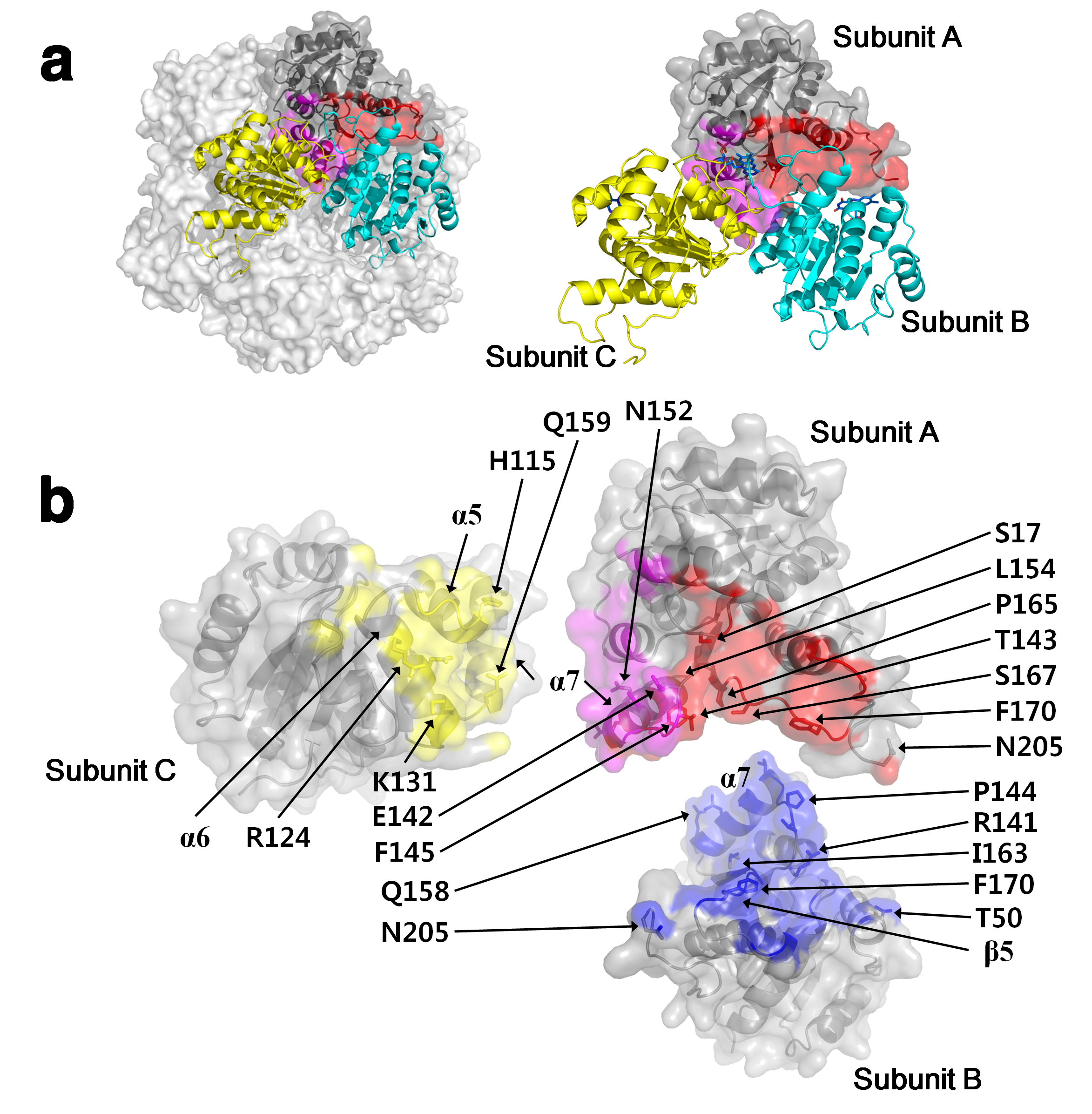


Figure S2. Intersubunit contact regions of FMN-bound (wild-type) CpsUbiX (a) The dodecamer structure of CpsUbiX showing the two patches of the subunit interface, indicated in red (patch 1) and magenta (patch 2). (b) The three subunits including the two patches of the subunit interface were separated to show the respective contact surfaces. The subunit B and C are tilted to improve the visualization of the contact area. The contact surfaces for subunit B and C are indicated in blue and yellow, respectively. The intersubunit contact residues are shown. The interface patch 1 is composed of 18 apolar residues, 12 hydrogen bonds, and 5 salt bridges, whereas the patch 2 involves 10 apolar residues, 9 hydrogen bonds, and 6 salt bridges. The intersubunit contacts were analyzed using the CCP4 programs CONTACT^9^ and LigPlot^8^.

Supplementary Table S1

Table S1. Residues involved in the inter-subunit contacts of the FMN-bound CpsUbiX structure

| **Patch 1** |  |  |  | **Patch 2** |  |  |
| --- | --- | --- | --- | --- | --- | --- |
| Subunit A | Subunit B | Distance (Å) |  | Subunit B | Subunit C | Distance (Å) |
| *Hydrophobic interactions* | | |  | *Hydrophobic interactions* | | |
| Pro144 CB | Thr162 CG2 | 3.9 |  | Ile46 CD1 | Trp86 CD1 | 3.8 |
| Pro144 CG | His191 CE1 | 3.9 |  | Ala111 CB | Gly116 C | 3.6 |
| Phe145 CE2 | Phe145 CE2 | 3.7 |  | Ala111 CB | Met117 CA | 4.2 |
| Leu150 CD2 | Leu 150 CD2 | 3.8 |  | His115 CD2 | Met117 CE | 3.5 |
| Leu150 CD | Met 153 CB | 3.9 |  | Leu148 CD1 | Gly116 CA | 3.7 |
| Leu154 CD1 | Gln151 CG | 3.7 |  | Leu148 CD2 | Ile130 CD1 | 4.1 |
| Leu154 CD2 | Leu154 CD2 | 3.7 |  | His149 CE1 | Lys131 CB | 4.1 |
| Pro165 CG | Phe145 CD2 | 3.4 |  |  |  |  |
| Phe170 CE1 | Tyr21 CE2 | 3.9 |  |  |  |  |
| Leu180 CD1 | Ile177 CG2 | 4.1 |  |  |  |  |
| Phe183 CE1 | Thr143 CG2 | 4.3 |  |  |  |  |
|  |  |  |  |  |  |  |
| *Polar interactions* | | |  | *Polar interactions* | | |
| Ser17 OG | Phe170 N | 2.8 |  | His115 O | Asn152 ND2 | 3.0 |
| Thr50 OG1 | Asn205 O | 2.6 |  | Arg124 NH1 | Glu142 OE1 | 2.9 |
| Arg141 NH2 | Ser167 O | 3.0 |  | Asp127 OD2 | His149 NE2 | 3.1 |
| Thr143 OG1 | Pro165 O | 2.7 |  | Lys131 NZ | Glu142 OE2 | 2.8 |
| Phe145 N | Ile163 O | 3.0 |  | Lys131 NZ | Thr 143 O | 3.1 |
| Thr147 OG1 | Ser157 OG | 2.7 |  | Glu132 OE1 | His191 NE2 | 2.6 |
| Ser157 OG | Thr147 OG1 | 2.8 |  | Glu132 OE2 | Arg187 NH1 | 2.8 |
| Ile163 O | Phe145 N | 3.0 |  | Arg133 NH1 | Gly160 O | 3.1 |
| Pro165 O | Thr143 OG1 | 2.7 |  | Gln159 NE2 | Gln151 OE1 | 3.0 |
| Ser167 O | Arg141 NH1 | 2.7 |  |  |  |  |
| Ser167 OG | Ser167 O | 2.6 |  |  |  |  |
| Phe170 N | Ser17 OG | 2.8 |  |  |  |  |

Supplementary Figure S3

Figure S3. Structural comparison of CpsUbiXs and AtHAL3 (PDB ID: 1MVN)^11^. (a) Ribbon diagram showing the trimer structures of CpsUbiX (V47S mutant, FMN-free), CpsUbiX / FMN (FMN-bound), and a monomer of AtHAL3 (FMN and PCO bound). The red colored loops in CpsUbiXs represent residues Phe170-Thr176. The blue colored regions in CpsUbiXs represent residues Glu195 to the C-terminus. Conformational differences of the loops are observed between FMN-free and FMN-bound CpsUbiXs. The red colored region of AtHAL3 represents residues Lys171-Asp177 and the loop folds itself to construct a substrate-binding site. Note the same loop in CpsUbiX is folded opposite direction. (b) Superposition of FMN-bound CpsUbiX and AtHAL3 showing the detailed view of substrate binding site of AtHAL3 and its relative position in CpsUbiX. CpsUbiX and AtHAL3 are depicted in orange and cyan, respectively. FMN and PCO are shown as sticks. All the proteins are in the same orientation.
